# PSA density for clinically significant prostate cancer detection: a retrospective cohort study on the influence of age and prostate volume

**DOI:** 10.1007/s00345-026-06613-9

**Published:** 2026-08-14

**Authors:** Lucas Engelage, Niklas Behnel, Agron Lumiani, Oleksii Bashkanov, Leo Federico Stadelmeier, Alexander Tamalunas, Alexander Buchner, Ronald Sroka

**Affiliations:** 1 Department of Urology, University Hospital, LMU Munich, Marchioninistr. 15, 81377 Munich, Germany; 2 ALTA Klinik, Bielefeld, Germany; 3 Laser-Forschungslabor, LIFE Center, University Hospital, LMU Munich, Munich, Germany; 4 Department of Anesthesiology and Intensive Care Medicine, Campus Charité Mitte and Campus Virchow-Klinikum, Charité – Universitätsmedizin Berlin, Berlin, Germany; 5 Comprehensive Cancer Center (CCC Munich, LMU), LMU University Hospital, Munich, Germany

**Keywords:** PSA density, Prostate-specific antigen, Prostate volume, Clinically significant prostate cancer, Diagnostic performance, ROC analysis

## Abstract

**Purpose:**

PSA density (PSAD) may improve detection of clinically significant prostate cancer (csPCa) by normalizing PSA for prostate volume. Current practice applies a uniform PSAD threshold of 0.15 ng/ml/cm³; however, whether this approach performs adequately across prostate volumes remains unclear. This study evaluated the diagnostic performance of PSAD for csPCa and investigated the influence of age and prostate volume on its accuracy.

**Methods:**

A total of 3,923 MRI-guided biopsy sessions from 3,087 patients were retrospectively analyzed. PSAD was calculated from total PSA (ng/ml) and MRI-derived prostate volume (cm³). ROC analysis determined AUC and Youden-optimal cut-offs, stratified by age (< 60, 60–69, ≥ 70 years) and prostate volume (< 30, 30–50, > 50 cm³), with bootstrap CIs.

**Results:**

Overall AUC was 0.758 (95% CI 0.743–0.773). Optimal cut-offs varied substantially by volume: 0.246 ng/ml/cm³ (95% CI 0.156–0.292) for small (< 30 cm³, AUC 0.717), 0.144 ng/ml/cm³ (0.124–0.178) for moderate (30–50 cm³, AUC 0.734), and 0.124 ng/ml/cm³ (0.102–0.162) for large prostates (> 50 cm³, AUC 0.702). Accuracy deteriorated progressively in very large prostates (AUC 0.658 for > 80 cm³, 0.609 for > 120 cm³). At volumes > 80 cm³, the 0.15 ng/ml/cm³ threshold achieved only 33.1% sensitivity. Age showed minimal clinical impact (AUC improvement 0.019).

**Conclusion:**

A universal 0.15 ng/ml/cm³ PSAD threshold fails to account for prostate volume variability. Potential volume-adapted cut-offs (0.25 / 0.15 / 0.12 ng/ml/cm³ for < 30 / 30–50 / >50 cm³) better approximate stratum-specific optimal values, although continuous multivariable risk prediction outperformed any fixed-threshold strategy and PSAD is interpreted alongside MRI in practice. Alternative biomarkers should be considered for very large prostates where PSAD accuracy deteriorates. These findings are hypothesis-generating and require external validation with PI-RADS integration before potential guideline implementation.

**Supplementary Information:**

The online version contains supplementary material available at 10.1007/s00345-026-06613-9.

## Introduction

Serum prostate-specific antigen (PSA) is a key marker in prostate cancer (PCa) detection, but its limited specificity leads to unnecessary biopsies for benign disease or indolent cancer. PSA levels can be elevated due to benign prostatic hyperplasia (BPH) or prostatitis, especially in men with larger glands [1]. To improve predictive accuracy, PSA density (PSAD), defined as the PSA level divided by prostate volume, was introduced in the early 1990 s [2]. The rationale is that higher PSAD indicates a disproportionately elevated PSA relative to gland size, raising suspicion for malignancy rather than benign enlargement. PSAD threshold values in the range of 0.10–0.15 ng/ml/cm³, with 0.15 ng/ml/cm³ being the most commonly applied, have been found predictive of clinically significant prostate cancer (csPCa, Gleason Grade Group ≥ 2) on biopsy [3–7], whereas a low PSAD is associated with very low risk of clinically significant disease [1, 8].

In the era of multiparametric MRI (mpMRI), PSAD has gained attention as an adjunct marker for biopsy decisions [7, 9, 10]. Current EAU guidelines recommend incorporating PSAD with MRI findings: for PI-RADS 3 lesions with PSAD < 0.10 ng/ml/cm³, biopsy may be safely deferred [3]. Recent studies demonstrate superior performance of PSAD over PSA alone (AUC 0.78 vs. 0.64) with optimal cutoff values of 0.15–0.20 ng/ml/cm³ depending on clinical context [11, 12].

However, it remains unclear whether a single cutoff value is appropriate for all patients. Both PSA and prostate volume increase with age, potentially affecting PSAD performance across subgroups [13]. Quarta et al. recently demonstrated that the accuracy of PSAD varies with prostate volume in an mpMRI-selected cohort [14]. As it is unknown, whether such findings can be extended to broader biopsy populations, there is need to evaluate the diagnostic performance of PSAD in a large contemporary biopsy cohort and to investigate whether age-specific or volume-specific cutoff values improve sensitivity and specificity compared to a uniform approach. We hypothesized that (a) prostate volume modifies PSAD accuracy more strongly than age, and (b) the conventional 0.15 ng/ml/cm³ cut-off systematically over-diagnoses csPCa in small glands and under-diagnoses it in large glands.

## Methods

### Study population

Data from men evaluated for suspected prostate cancer between 2012 and 2022 were retrospectively analyzed. The study was conducted as a retrospective, anonymized analysis of routinely acquired clinical MRI and biopsy data. According to the Ethics Committee of the Medical Faculty and the Medical Association of Westphalia-Lippe (reference number 2014-007-f-N), ethical approval was waived in view of the retrospective nature of the study and all procedures being part of routine clinical care. This study was conducted in accordance with the principles of the Declaration of Helsinki. Inclusion criteria comprised elevated PSA and/or abnormal digital rectal examination prompting further diagnostic work-up, availability of pre-biopsy MRI-derived prostate volume, and either histopathological confirmation from prostate biopsy or PI-RADS 1–2 imaging with at least 12 months of clinical follow-up without subsequent csPCa diagnosis. Men with a history of prior treatment for prostate cancer were excluded. For patients with repeat evaluations, the minimum interval between two biopsy sessions was 6 months. A total of 3,087 patients contributed 3,923 independent observations (2,713 single evaluations, 374 with multiple assessments during active surveillance or repeat biopsy).

### MRI and biopsy procedures

Prostate MRI was performed either at ALTA Klinik, Bielefeld, Germany (Siemens MAGNETOM Avanto 1.5T and Skyra 3 T, Siemens Healthineers, Erlangen, Germany) or at external centers (various 1.5T and 3 T scanners from multiple vendors with heterogeneous protocols). Prostate volume was determined on axial T2-weighted sequences by manual planimetry using the ellipsoid formula (V = 4/3 × π × a × b × c, where a, b, and c denote the semi-axes measured in three orthogonal planes) in the Medigration PACS Viewer (medigration GmbH, Baden-Baden, Germany). Each examination was reviewed by at least two readers, with consensus review in cases of disagreement. Serum total PSA was measured within one week of imaging using various commercially available immunoassays at referring laboratories; specific assay information was not systematically recorded. PSAD was calculated as PSA (ng/ml) divided by MRI-derived prostate volume (cm³). MRI-fusion-guided biopsies were performed using a 12-core systematic template supplemented by 2–5 targeted cores from each PI-RADS 3–5 lesion; both transrectal and transperineal approaches were used according to operator preference and patient anatomy. Biopsy pathology results were recorded, including Gleason score, presence of high-grade prostatic intraepithelial neoplasia or intraductal carcinoma, and significant chronic inflammation in benign tissue.

### Outcome definition

The primary outcome was csPCa, defined as ISUP Grade Group ≥ 2 (Gleason ≥ 3 + 4) [3]. Grade Group 1, benign biopsy findings, and PI-RADS 1–2 patients without biopsy who remained free of csPCa during at least 12 months of follow-up were classified as “no csPCa”.

### Statistical analysis

Continuous variables were summarized as medians with interquartile ranges (IQR) and compared using the Mann–Whitney U test; categorical variables were compared using the chi-square test. The diagnostic performance of PSAD was evaluated by receiver operating characteristic (ROC) curve analysis with Youden-optimal cut-off values [15]. Prostate volume was a priori categorized as prostate volume < 30 cm³ (small), 30–50 cm³ (moderate), and > 50 cm³ (large) based on established clinical conventions [16]; additional subgroups of very large prostates (> 80, > 100, >120 cm³) and age strata (< 60, 60–69, ≥ 70 years) were exploratory. The chosen primary strata reflect a balance between clinical interpretability and stratum-level sample size. Given the multiple primary, exploratory, age, and very-large-volume strata evaluated across two models without correction for multiple comparisons, p-values and point estimates from the exploratory strata — particularly the small > 100 cm³ (*n* = 317) and > 120 cm³ (*n* = 159) subgroups — are reported as descriptive and hypothesis-generating rather than confirmatory, and are presented to illustrate trends rather than to support formal inference.

Two pre-specified multivariable logistic regression models evaluated age as a potential confounder: Model 1 included PSAD and prostate volume; Model 2 additionally included age. AUC differences between models were tested by bootstrap resampling. Several sensitivity and exploratory analyses are reported in detail in the Online Resource: internal validation by patient-clustered bootstrap optimism correction (S1); first-biopsy-only re-derivation and cluster-robust standard errors for repeated measures (S2); formal testing of the PSAD × volume interaction by B-spline logistic regression (S3); decision curve analysis (S4); chronic prostatitis as a multivariable covariate with prostatitis-excluded sensitivity analysis (S5). No formal correction for multiple testing was applied. Analyses were performed in R (version 4.4.1, R Foundation for Statistical Computing, Vienna, Austria) using the packages stats and pROC, and in Python (version 3.12, Python Software Foundation) using statsmodels, scikit-learn, scipy, and patsy. All tests were two-sided with *p* < 0.05 considered significant.

## Results

### Patient demographics and baseline characteristics

A total of 3,923 MRI-guided biopsy sessions from 3,087 patients were analyzed (Table 1). csPCa was identified in 1,635 sessions (41.7% of all sessions, 65.0% of detected cancers), comprising Grade Group 2 in 842 cases (51.5%), Grade Group 3 in 336 (20.6%), and Grade Group 4–5 in 457 (27.9%). csPCa patients showed approximately twofold higher PSAD than non-csPCa patients (median 0.20 vs. 0.11 ng/ml/cm³, *p* < 0.001), driven by both higher PSA and smaller prostate volume.

**Table 1:** Patient demographics and clinical characteristics stratified by presence of clinically significant prostate cancer (csPCa, ISUP Grade Group ≥ 2)

Characteristic	Non-csPCa (*n* = 2,288)	csPCa (*n* = 1,635)	*p*-value
Age (years)	65 (60–71)	68 (63–74)	< 0.001
PSA (ng/ml)	6.2 (4.2–8.8)	7.9 (5.5–12.2)	< 0.001
PSAD (ng/ml/cm³)	0.11 (0.07–0.16)	0.20 (0.13–0.32)	< 0.001
Chronic prostatitis	1,167 (51.0%)	382 (23.4%)	< 0.001
Unbiopsied (MRI-negative)	205 (9.0%)	–	–
No cancer (benign)	1,407 (61.5%)	0 (0%)	–
Gleason 6 (GG 1)	881 (38.5%)	0 (0%)	–
Gleason 7 (3 + 4) – GG 2	0 (0%)	842 (51.5%)	–
Gleason 7 (4 + 3) – GG 3	0 (0%)	336 (20.6%)	–
Gleason ≥ 8 – GG 4–5	0 (0%)	457 (27.9%)	–
Prostate volume (cm³)	53.0 (38.1–76.4)	39.5 (30.4–54.6)	< 0.001
Prostate volume < 30 cm³	229 (10.0%)	394 (24.1%)	< 0.001
Prostate volume 30–50 cm³	825 (36.1%)	737 (45.1%)	< 0.001
Prostate volume > 50 cm³	1,234 (53.9%)	504 (30.8%)	< 0.001

*C*ontinuous variables are presented as median (interquartile range) and compared using the Mann–Whitney U test. Categorical variables are presented as number (percentage) and compared using the χ² test. Unbiopsied patients were MRI-negative (PI-RADS 1–2) and classified as “no csPCa” based on defined negative clinical follow-up

**Fig. 1:**
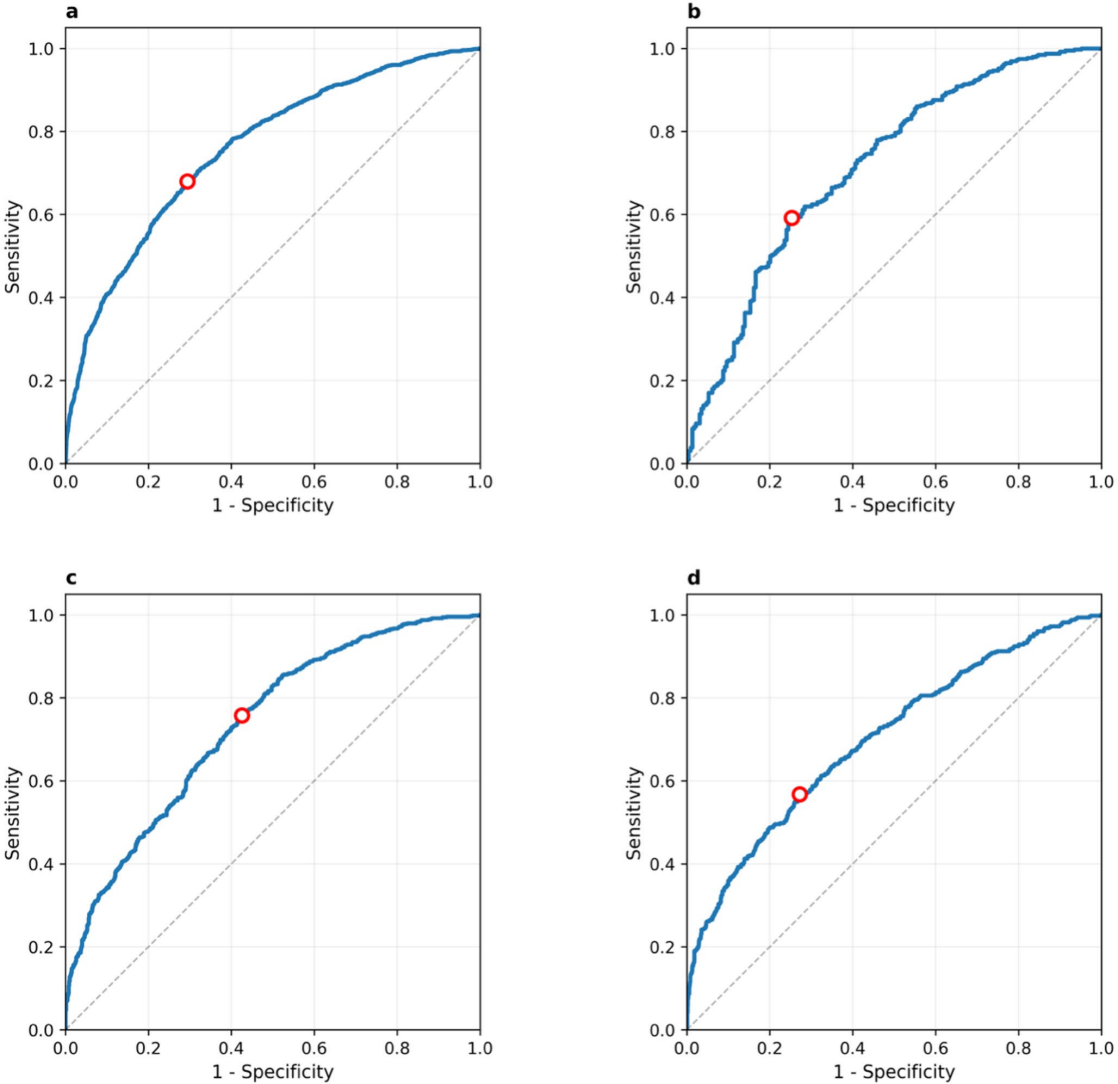
Receiver operating characteristic (ROC) curves of PSAD for detecting clinically significant prostate cancer (csPCa, ISUP Grade Group ≥ 2), stratified by prostate volume. **a** Entire cohort (AUC 0.758; Youden-optimal cutoff value 0.149 ng/ml/cm³; sensitivity 69.1%, specificity 69.2%). **b** Small prostates (Vp < 30 cm³; AUC 0.717; cutoff value 0.246 ng/ml/cm³; sensitivity 59.1%, specificity 74.7%). **c** Medium prostates (Vp = 30–50 cm³; AUC 0.734; cutoff value 0.144 ng/ml/cm³; sensitivity 75.8%, specificity 57.5%). **d** Large prostates (Vp > 50 cm³; AUC 0.702; cutoff value 0.124 ng/ml/cm³; sensitivity 56.7%, specificity 72.8%). Red circles indicate Youden-optimal cutoff values

### PSAD in the overall cohort

Overall, PSAD discriminated csPCa with an AUC of 0.758 (95% CI 0.743–0.773; Fig. 1a). The Youden-optimal cut-off of 0.149 ng/ml/cm³ (bootstrap 95% CI 0.124–0.171) achieved 69.1% sensitivity and 69.2% specificity, correctly identifying 1,129 of 1,635 csPCa cases at the cost of 706 false-positive results. To address within-patient correlation from repeated sessions, the analysis was repeated restricting to first-biopsy sessions only and using multivariable models with cluster-robust standard errors; both yielded materially unchanged discrimination and optimal cut-offs (Online Resource S2), indicating that the repeated-measures structure did not drive the results.

### PSAD in volume-stratified analyses

Volume-stratified analysis revealed pronounced differences in optimal cut-offs and discriminative performance (Fig. 1b–d). For small prostates (< 30 cm³), the Youden-optimal cut-off was 0.246 ng/ml/cm³ (bootstrap 95% CI 0.156–0.292; AUC 0.717; sensitivity 59.1%, specificity 74.7%). For moderate prostates (30–50 cm³), the optimal cut-off was 0.144 ng/ml/cm³ (0.124–0.178; AUC 0.734; sensitivity 75.8%, specificity 57.5%). For large prostates (> 50 cm³), the optimal cut-off was 0.124 ng/ml/cm³ (0.102–0.162; AUC 0.702; sensitivity 56.7%, specificity 72.8%).

Applied within each stratum, the conventional fixed 0.15 ng/ml/cm³ cut-off showed strongly volume-dependent performance: in small prostates, sensitivity was 87.1% but specificity only 40.6%; in moderate prostates, 72.2% and 60.1%; in large prostates, 44.8% and 83.1% (Online Resource S1). Continuous modeling of csPCa probability across PSAD and prostate volume confirmed the volume-dependency of the optimal PSAD threshold (Online Resource S3). A formal PSAD × volume interaction was significant in B-spline logistic regression (Online Resource S3). In decision curve analysis the continuous risk prediction derived from the multivariable model (PSAD and prostate volume) yielded a higher net benefit than either the fixed 0.15 ng/ml/cm³ threshold or the volume-adapted triplet (0.25/0.15/0.12 ng/ml/cm³) across all clinically relevant threshold probabilities, making continuous risk the strongest-performing strategy and any fixed threshold a simplification of it (Online Resource S4).

### PSAD in age-stratified analyses

Age-stratified analyses demonstrated comparable diagnostic performance of PSAD across age groups (AUC range 0.755–0.784). Optimal cut-offs varied only modestly between strata (0.131–0.149 ng/ml/cm³). Adding age to PSAD and prostate volume in the multivariable model (Model 2 vs. Model 1) increased AUC from 0.764 to 0.783 (ΔAUC 0.019, 95% CI 0.011–0.027, *p* < 0.001); age contributed a modest independent effect (OR 1.32 per 5-year increment, *p* < 0.001).

**Fig. 2:**
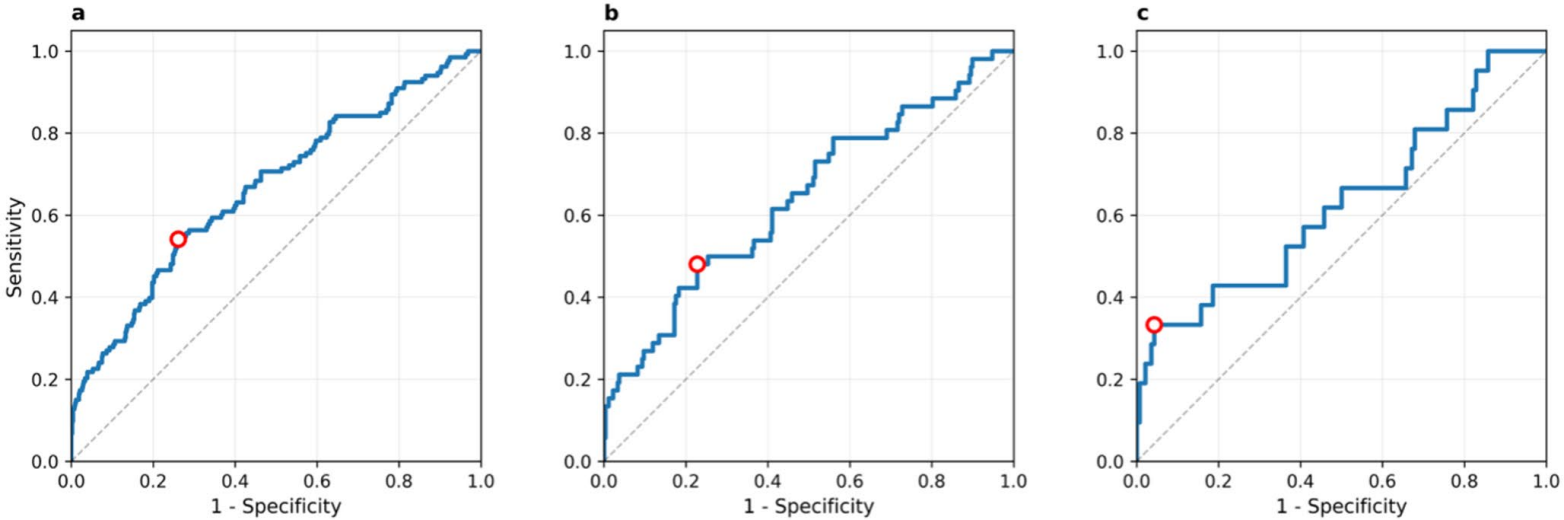
ROC curves of PSAD for detecting csPCa in patients with very large prostate volumes. **a **Vp > 80 cm³ (*n* = 663; AUC 0.658; Youden-optimal cutoff value 0.11 ng/ml/cm³; sensitivity 55.9%, specificity 69.4%). **b **Vp > 100 cm³ (*n* = 317; AUC 0.627; cutoff value 0.13 ng/ml/cm³; sensitivity 38.8%, specificity 82.5%). **c **Vp > 120 cm³ (*n* = 159; AUC 0.609; cutoff value 0.24 ng/ml/cm³; sensitivity 31.6%, specificity 95.7%). Red circles indicate Youden-optimal cutoff values

### Very large prostates and clinical trade-offs

Detailed analysis of very large prostates demonstrated progressive deterioration of diagnostic accuracy (Fig. 2). For volumes > 80 cm³ (*n* = 663, csPCa prevalence 19.2%), the conventional 0.15 ng/ml/cm³ threshold achieved only 33.1% sensitivity; the Youden-optimal cut-off was 0.11 ng/ml/cm³ sensitivity 55.9%, specificity 69.4%, AUC 0.658). For volumes > 100 cm³ (*n* = 317, csPCa 15.5%), AUC decreased to 0.627 (optimal cut-off 0.13 ng/ml/cm³, sensitivity 38.8%, specificity 82.5%). For volumes > 120 cm³ (*n* = 159, csPCa 11.9%), AUC further declined to 0.609 (optimal cut-off 0.24 ng/ml/cm³, sensitivity 31.6%, specificity 95.7%).

Decision curve analysis quantified the trade-offs: relative to the fixed 0.15 cut-off, the volume-stratified strategy (0.25/0.15/0.12 ng/ml/cm³) avoided 196 biopsies but missed 116 csPCa in small prostates, was equivalent in moderate prostates, and detected 66 additional csPCa for 230 additional biopsies in large prostates; continuous risk prediction from the multivariable model dominated both threshold-based strategies across all examined threshold probabilities (Online Resource S4).

## Discussion

PSAD is widely used to refine total PSA, yet most clinical pathways apply fixed thresholds (commonly 0.15 ng/ml/cm³), implicitly assuming comparable performance across prostate phenotypes. Consistent with prior studies reporting AUCs of 0.68–0.78 [1, 7, 11], PSAD provided superior discrimination for csPCa compared to PSA alone in the present cohort. However, our subgroup analyses reveal systematic, volume-dependent misclassification that aligns with recent critiques [9, 17] and is not currently acknowledged in EAU [18] or German S3 [19] guideline frameworks, which apply PI-RADS-conditional PSAD thresholds (in the current EAU framework, PSAD < 0.10 ng/ml/cm³ to defer biopsy for PI-RADS 3 lesions and < 0.20 ng/ml/cm³ for negative MRI [PI-RADS ≤ 2]) uniformly across prostate volumes within each PI-RADS category.

### Age-dependent performance

While age reached statistical significance in multivariable analysis (OR 1.32 per 5-year increment, *p* < 0.001), the AUC improvement of 0.019 from adding age to a model already containing PSAD and prostate volume is unlikely to affect clinical decision-making. Combined with consistent PSAD performance across age strata (AUC 0.755–0.784), this indicates that prostate volume rather than age should guide cut-off selection, aligning with evidence that age-related PSA changes are primarily mediated through prostatic enlargement [13].

### Volume-dependent performance

The central finding of the present study is that prostate volume dependency transforms PSAD from a uniform diagnostic marker into a source of systematic, phenotype-dependent decision-making error. For small prostates, the optimal cut-off of 0.246 ng/ml/cm³ was substantially higher than the conventional threshold. Robinson et al. corroborated this volume-dependent gradient, reporting 83.1% sensitivity in small versus 33.3% in large prostates at the 0.15 ng/ml/cm³ threshold [20]. Conflicting earlier reports — Pellegrino et al. proposing ≥ 0.20 ng/ml/cm³ [21] and Nordström et al. reporting only 49% csPCa detection at 0.15 [7] — are reconcilable through differences in cohort volume distribution, as shown by Quarta et al., whose data demonstrated that csPCa risk at PSAD 0.10 ng/ml/cm³ varied from 37% (< 45 cm³) to 15% (60–100 cm³) [14], and by Bostancı et al., who reported TRUS-based detection rates of 78.5% in prostates ≤ 35 cm³ versus 17.2% in ≥ 76 cm³ at comparable PSAD values [22]. Differences in cohort composition (biopsy-naïve versus repeat biopsy, MRI-based versus TRUS-based volumetry, screening versus clinical suspicion) likely contribute to the heterogeneity in published optimal cut-offs and reinforce the case for adapted rather than universal thresholds. Across this growing body of work — including biopsy-naïve MRI cohorts [20], mpMRI-selected populations [14, 21], and screening settings [7] — volume-adapted PSAD interpretation is emerging as a more refined alternative to a single threshold. Our cohort, spanning biopsy-naïve, active surveillance, and repeat-biopsy sessions, adds further support to this trajectory and allows the trade-offs to be quantified explicitly: decision curve analysis (Online Resource S4) confirms that the volume-adapted strategy in small prostates favors specificity over sensitivity — an acceptable balance in this high-prevalence phenotype where overdiagnosis is the primary concern raised by the S3 guideline [19], and where cut-offs as high as 0.30 ng/ml/cm³ have been proposed for benign prostatic obstruction [8].

In large prostates, the diagnostic performance of PSAD deteriorated substantially (AUC 0.702 for > 50 cm³, declining to 0.609 for > 120 cm³), and even the volume-adapted cut-off of 0.124 ng/ml/cm³ still missed 43.3% of csPCa. Decision curve analysis quantified the practical trade-off in this stratum as 66 additional csPCa detected at the cost of 230 additional biopsies (3.5 additional biopsies per additional csPCa; Online Resource S4) — a modest improvement over the fixed 0.15 cut-off, which itself systematically failed in this volume range (33.1% sensitivity at > 80 cm³). Continuous risk prediction from the multivariable model dominated both threshold-based strategies across the examined range of threshold probabilities, suggesting that the strongest gains in this volume range may lie not in revised cut-offs but in a shift towards probabilistic, multivariable decision rules. Omri et al. similarly observed PSAD’s loss of predictive value above 75 cm³, with sensitivity collapsing from 72.7% to 3.2% [23]. This deterioration reflects BPH pathophysiology: a 37-fold increase in stromal proliferation compared to normal prostate tissue dilutes PSA — produced exclusively by epithelial cells — relative to total volume, progressively undermining PSAD’s discriminatory capacity [24].

### Clinical implications

Translating these findings into practice does not require abandoning MRI-based pathways but rather refining how PSAD is interpreted within them. The volume-adapted cut-offs (0.25/0.15/0.12 ng/ml/cm³ for < 30/30–50/>50 cm³) approximate the Youden-optimal values within the bounds of assay variability and represent a low-cost candidate modification to existing decision rules; however, they should be regarded as potential thresholds rather than clinically actionable decision rules. Two caveats constrain their direct use. First, because PSAD in contemporary pathways is interpreted together with the PI-RADS category — and PI-RADS 4–5 lesions are biopsied irrespective of PSAD — these standalone cut-offs require integration with MRI before deployment and are most relevant to the equivocal PI-RADS 3 setting. Second, decision curve analysis indicated that continuous multivariable risk prediction outperformed both the fixed and the volume-adapted fixed thresholds, suggesting that the principal gain lies in moving toward probabilistic risk estimation rather than in substituting one fixed threshold for another. Beyond pre-biopsy stratification, PSA-derived parameters retain residual predictive value in large-volume contexts where PSAD itself becomes structurally unreliable: Tsaur et al. showed that pre-operative PSA independently predicted unfavorable pathology in incidental PCa diagnosed at BPO surgery [25], a setting where prostate volume is by definition large enough to mandate surgical intervention. In very large prostates (> 80 cm³), neither EAU [18] nor German S3 guidelines [19] provide specific recommendations; alternative biomarkers such as the Stockholm3 test [26], PHI density (AUC 0.826) [27], or 4 K Density [28] may enable meaningful risk stratification where PSAD assessment fails. These findings are hypothesis-generating; prospective multicenter validation with standardized PSA assays, MRI and TRUS volumetry side-by-side, and integration of PI-RADS with adjunct biomarkers is required before guideline implementation.

### Strengths and limitations

Strengths include the large sample size, the inclusion of active surveillance and repeat-biopsy sessions reflecting real-world clinical heterogeneity, and the use of MRI-derived volumes consistent with current best practice in volumetric assessment. This heterogeneity is also a limitation: the cohort combines biopsy-naïve, repeat-biopsy, and active-surveillance sessions, which differ in baseline risk, pre-test probability, biopsy indication, and the clinical interpretation of PSAD, and these distinct populations could not be cleanly separated for stratified diagnostic analysis in the retrospective dataset. The volume-dependent behaviour of PSAD reported here should therefore be read as an aggregate effect across these groups rather than as established within each clinically distinct population; confirming consistency across biopsy-naïve, repeat-biopsy, and active-surveillance settings is a task for prospective data. Likewise, detailed pathological characterisation of the Grade Group 2 subgroup — proportion of Gleason pattern 4, number and proportion of positive cores, maximum cancer core length, and the share meeting favourable-risk or active-surveillance criteria — was not systematically available in the source dataset and could not be reported, so minimal-volume Grade Group 2 disease cannot be distinguished here from more extensive csPCa.

Limitations include the retrospective single-centre design and a high-volume MRI-guided cohort with csPCa prevalence (41.7%) that is not generalizable to screening or primary care populations; prevalence-dependent metrics such as PPV/NPV should be interpreted accordingly. The 205 PI-RADS 1–2 patients classified as no-csPCa based on ≥ 12 months of clinical follow-up rather than histopathology (5.2% of sessions) constitute a potential source of outcome misclassification; latent csPCa missed by MRI and clinically silent over this interval would bias diagnostic estimates towards the null. Repeated biopsy sessions in 374 patients introduce within-patient correlation that may underestimate standard errors in conventional analyses; sensitivity analyses restricted to first-biopsy sessions and multivariable models with cluster-robust standard errors (Online Resource S2) suggest that diagnostic estimates are not materially affected.

Imaging was acquired across heterogeneous scanners and protocols without centralized re-read or formal inter-rater agreement statistics, introducing potential measurement variability. PSA was measured at referring laboratories using various commercially available immunoassays; the resulting inter-assay variability of 20–30% [29–31] limits the precision of cut-off values. Further, MRI-derived volumes are not directly interchangeable with TRUS-based volumes — Choe et al. reported underestimation of 8–39% depending on gland size and reclassification of 22.5% of patients below the 0.15 PSAD threshold when switching to MRI [32] — and the proposed volume-adapted cut-offs therefore require recalibration before extrapolation to TRUS-based settings, where superior MRI reproducibility (inter-rater ICC 0.91 versus 21.9% inter-observer variability for TRUS) further favors MRI-based assessment [33–35].

The diagnostic model deliberately focused on PSAD and prostate volume and did not incorporate PI-RADS lesion characteristics, which were not systematically documented in the source dataset; contemporary MRI-based pathways combine PSAD with PI-RADS for biopsy decisions, and the present analysis therefore likely overstates the standalone contribution of PSAD. A meta-analysis of 36,366 patients showed that combined PSAD-MRI approaches avoided 30–48% of unnecessary biopsies while missing 3–5% of csPCa [36]. A structural reason underlies the absence of PI-RADS stratification: the cohort opened in 2012, before PI-RADS v2 (2015) and its subsequent revisions established the contemporary five-category standard, so historically consistent PI-RADS scores were not retrievable across the full study period. The proposed cut-offs are therefore not directly deployable as stand-alone rules and require integration with MRI before clinical use, most pertinently in the equivocal PI-RADS 3 setting where PSAD currently informs the biopsy decision. This positions the present work as a description of the standalone, volume-dependent behaviour of PSAD that is complementary to, rather than a substitute for, PI-RADS-stratified analyses. Contemporary series that do stratify by PI-RADS report the same volume gradient while refining PSAD thresholds by imaging category and index-lesion location: Quarta et al. showed that the association between PSAD and csPCa differs by index-lesion zone, with PI-RADS 3 lesions reaching clinically relevant csPCa risk only above PSAD ≈ 0.05 ng/ml/cm³ (peripheral zone) and ≈ 0.13 ng/ml/cm³ (transition zone), whereas PI-RADS 4–5 lesions carried non-negligible risk regardless of PSAD [37]; and Nazir et al. confirmed in PI-RADS 3 lesions that the 0.15 ng/ml/cm³ threshold balances sensitivity and specificity within that category [38]. Integrating volume-adapted PSAD with PI-RADS category in prospective data is the necessary next step. Finally, the proposed volume-adapted cut-offs have not been tested in an independent cohort, and external validation in prospective multicenter data — particularly in TRUS-based or screening populations — is required before incorporation into guideline recommendations.

## Conclusion

Fixed PSAD thresholds introduce systematic misclassification — overdiagnosis in small prostates and underdiagnosis in large prostates — that undermines guideline objectives. The proposed volume-adapted cut-offs (0.25/0.15/0.12 ng/ml/cm³ for < 30/30–50/>50 cm³) should be regarded as potential thresholds rather than clinically actionable decision rules, since PI-RADS 4–5 lesions are biopsied regardless of PSAD and continuous multivariable risk prediction outperformed any fixed-threshold strategy; their main role lies in the equivocal PI-RADS 3 setting and requires integration with MRI. In very large prostates (> 80 cm³), where PSAD-based risk assessment becomes structurally unreliable, alternative biomarkers such as PHI density or 4 K Density warrant consideration. These findings are hypothesis-generating and require prospective multicenter validation — with standardized PSA assays and harmonized MRI/TRUS volumetry — before incorporation into guideline recommendations.

## Supplementary Information

Below is the link to the electronic supplementary material.

Supplementary Material 1

## Data Availability

The datasets generated and analyzed during the current study are available from the corresponding author on reasonable request.
