# Supplementary material for: PSA density for clinically significant prostate cancer detection: a retrospective cohort study on the influence of age and prostate volume
Source: World J Urol. 2026 Aug 14;44(1):565. doi: 10.1007/s00345-026-06613-9 (PMC13476352; doi:10.1007/s00345-026-06613-9)
Supplement: Supplementary file 1 [file 345_2026_6613_MOESM1_ESM.docx]

**Supplementary Statistical Analyses**

*Online Resource — Detailed Methods and Results*

# S1 — Internal validation by bootstrap optimism correction

### Methods

Optimism-corrected estimates of diagnostic performance were obtained using B = 2,000 patient-clustered bootstrap iterations. In each iteration, 3,087 patients were sampled with replacement together with all their sessions; Youden-optimal PSAD cut-offs were re-derived on the bootstrap sample and then evaluated on the original cohort. Optimism was computed as the mean difference between in-sample and out-of-sample AUC. Optimism-corrected AUC is reported as apparent AUC minus optimism. Bootstrap 95 % confidence intervals for cut-offs, sensitivity and specificity were derived from the 2.5th to 97.5th percentiles of the bootstrap distribution.

### Results

Optimism-corrected diagnostic performance per volume stratum (Table S1):

| **Stratum** | **n** | **csPCa (%)** | **Apparent AUC** | **Optimism** | **Corrected AUC (95 % CI)** | **Cut-off (95 % CI)** |
| --- | --- | --- | --- | --- | --- | --- |
| Overall | 3,923 | 1,635 (41.7) | 0.758 | −0.0000 | 0.758 (0.739–0.777) | 0.149 (0.124–0.171) |
| <30 cm³ | 623 | 394 (63.2) | 0.717 | −0.0005 | 0.718 (0.660–0.773) | 0.246 (0.156–0.292) |
| 30–50 cm³ | 1,562 | 737 (47.2) | 0.734 | −0.0000 | 0.734 (0.703–0.764) | 0.144 (0.124–0.178) |
| >50 cm³ | 1,738 | 504 (29.0) | 0.702 | +0.0002 | 0.702 (0.669–0.736) | 0.124 (0.102–0.162) |
| >80 cm³ | 663 | 127 (19.2) | 0.658 | +0.0007 | 0.658 (0.604–0.724) | 0.112 (0.087–0.129) |
| >100 cm³ | 317 | 49 (15.5) | 0.627 | +0.0016 | 0.627 (0.545–0.734) | 0.131 (0.072–0.241) |
| >120 cm³ | 159 | 19 (11.9) | 0.609 | −0.0001 | 0.609 (0.469–0.768) | 0.241 (0.040–0.317) |

***Table S1.*** *Apparent and bootstrap optimism-corrected diagnostic performance of PSA density for csPCa detection, stratified by prostate volume. Cut-offs are Youden-optimal.*

Bootstrap optimism was ≤ 0.002 in every stratum, indicating minimal overfitting. The apparent and corrected AUC estimates were therefore practically equivalent.

### Performance of the conventional fixed PSAD ≥ 0.15 cut-off per volume stratum

For direct comparison with the volume-stratified strategy, sensitivity and specificity of the conventional fixed PSAD ≥ 0.15 ng/ml/cm³ threshold were computed within each stratum (Table S2):

| **Stratum** | **n** | **Sensitivity (%)** | **Specificity (%)** |
| --- | --- | --- | --- |
| <30 cm³ | 623 | 87.1 | 40.6 |
| 30–50 cm³ | 1,556 | 72.2 | 60.1 |
| >50 cm³ | 1,779 | 44.8 | 83.1 |
| >80 cm³ | 663 | 33.1 | 86.4 |
| >100 cm³ | 317 | 28.8 | 87.7 |
| >120 cm³ | 159 | 33.3 | 89.3 |

***Table S2.*** *Sensitivity and specificity of the fixed PSAD ≥ 0.15 ng/ml/cm³ cut-off within each volume stratum.*

Sensitivity of the fixed cut-off declines steeply with increasing prostate volume — from 87.1 % below 30 cm³ to 33.1 % above 80 cm³ — while specificity rises in parallel. This reciprocal pattern motivates the volume-stratified strategy: lower PSAD thresholds preserve sensitivity in large glands, and higher thresholds reduce unnecessary biopsies in small glands.

***Figure S1.*** *Bootstrap distribution of Youden-optimal PSAD cut-offs per volume stratum (B = 2,000 patient-clustered iterations). The red dashed line marks the apparent (point-estimate) cut-off; the black dotted lines indicate the 2.5th and 97.5th percentiles of the bootstrap distribution. Sample sizes per panel: Overall n = 3,923 (csPCa = 1,635); <30 cm³ n = 623 (csPCa = 394); 30–50 cm³ n = 1,556 (csPCa = 737); >50 cm³ n = 1,779 (csPCa = 504); >80 cm³ n = 663 (csPCa = 127); >100 cm³ n = 317 (csPCa = 49); >120 cm³ n = 159 (csPCa = 19).*

# S2 — Repeated measures sensitivity: first-biopsy subset

### Methods

Of 3,087 unique patients in the cohort, 374 contributed more than one biopsy session. To quantify any bias introduced by within-patient correlation, two complementary analyses were performed. First, diagnostic performance was re-derived on the chronologically first session per patient only (n = 3,087). Second, a random session per patient was repeatedly drawn for B = 1,000 iterations to obtain stability estimates for both AUC and Youden cut-off. In addition, all multivariable models reported elsewhere in this Supplement use cluster-robust standard errors with patient identifier as the clustering variable.

### Results

Comparison of full cohort vs. first-biopsy-only diagnostic performance (Table S3):

| **Stratum** | **Full n** | **First-bx n** | **Full AUC** | **First-bx AUC (95 % CI)** | **Δ AUC** | **Full cut-off** | **First-bx cut-off (95 % CI)** |
| --- | --- | --- | --- | --- | --- | --- | --- |
| Overall | 3,923 | 3,087 | 0.758 | 0.774 (0.771–0.777) | +0.018 | 0.149 | 0.141 (0.127–0.144) |
| <30 cm³ | 623 | 494 | 0.717 | 0.727 (0.717–0.736) | +0.019 | 0.246 | 0.244 (0.237–0.249) |
| 30–50 cm³ | 1,556 | 1,250 | 0.734 | 0.760 (0.755–0.766) | +0.029 | 0.144 | 0.139 (0.124–0.176) |
| >50 cm³ | 1,779 | 1,364 | 0.702 | 0.712 (0.707–0.718) | +0.012 | 0.124 | 0.128 (0.116–0.142) |
| >80 cm³ | 669 | 497 | 0.666 | 0.675 (0.663–0.685) | +0.015 | 0.112 | 0.112 (0.110–0.113) |
| >100 cm³ | 320 | 235 | 0.642 | 0.656 (0.637–0.673) | +0.030 | 0.131 | 0.109 (0.072–0.124) |
| >120 cm³ | 161 | 113 | 0.628 | 0.647 (0.608–0.685) | +0.055 | 0.241 | 0.240 (0.241–0.241) |

***Table S3.*** *Diagnostic performance derived from the full cohort compared to the chronologically first-biopsy-only subset per patient.*

Differences in AUC between full cohort and first-biopsy subset were ≤ 0.06 in every stratum; Youden cut-offs differed by ≤ 0.02 ng/ml/cm³ in all but the smallest subgroups. Within-patient correlation therefore introduces no meaningful bias in the reported diagnostic estimates.

# S3 — Formal testing of PSAD × prostate volume interaction

### Methods

To formally test whether the diagnostic effect of PSAD is modified by prostate volume, a hierarchical sequence of logistic regression models was fitted with cluster-robust standard errors (patient identifier as cluster). PSAD was log-transformed and standardised to zero mean and unit variance. Prostate volume was modelled both linearly and with B-splines (df = 4, patsy implementation) to capture potential non-linearity. Nested model comparisons used the likelihood-ratio test (LRT):

- Model A: csPCa ~ log_PSAD_std + volume
- Model B: csPCa ~ log_PSAD_std × volume (adds linear interaction)
- Model C-no-int: csPCa ~ log_PSAD_std + bs(volume, df = 4)
- Model C: csPCa ~ log_PSAD_std + bs(volume, df = 4) + log_PSAD_std : bs(volume, df = 4)
- Model D: Model C + age (per year)

### Results

Model fit statistics (Table S4):

| **Model** | **df** | **LogLik** | **AIC** | **Δ AIC vs D** |
| --- | --- | --- | --- | --- |
| A — linear, no interaction | 2 | −2,240.6 | 4,487.2 | +160.9 |
| B — linear + interaction | 3 | −2,240.1 | 4,488.3 | +162.0 |
| C-no-int — log + spline volume | 5 | −2,234.1 | 4,480.2 | +153.9 |
| C — + non-linear interaction | 9 | −2,222.7 | 4,465.4 | +139.1 |
| D — Model C + age | 10 | −2,152.1 | 4,326.3 | 0.0 |

***Table S4.*** *Fit statistics for the hierarchical sequence of logistic regression models. All models use cluster-robust standard errors with patient identifier as the clustering variable.*

Likelihood-ratio tests for nested model comparisons (Table S5):

| **Comparison** | **χ² (df)** | **p-value** | **Interpretation** |
| --- | --- | --- | --- |
| A → B (linear interaction) | 0.96 (1) | 0.327 | Linear interaction not statistically significant |
| C-no-int → C (non-linear interaction) | 22.76 (4) | 0.00014 | Non-linear interaction statistically significant |
| B → C (non-linearity beyond linear) | 34.88 (6) | < 0.001 | Non-linear structure improves fit over linear interaction |
| C → D (age contribution) | 141.11 (1) | < 0.001 | Age contributes significantly; ΔAUC = +0.019 |

***Table S5.*** *Likelihood-ratio tests across the hierarchical model sequence.*

The linear PSAD × volume interaction term (Model B) did not improve fit over the additive Model A. Modelling volume non-linearly with B-splines and allowing a non-linear interaction (Model C) produced a statistically significant improvement (χ²(4) = 22.76, p = 0.00014). Adding age (Model D) further improved fit (χ²(1) = 141.11, p < 0.001) but contributed only modestly to discrimination (ΔAUC = +0.020).

Model discrimination: Model C AUC = 0.7702; Model D AUC = 0.7903 (ΔAUC = +0.020). Age odds ratio per 5-year increment = 1.336 (Model D). Full model coefficients with cluster-robust standard errors are available in the supplementary code repository.

***Figure S2.*** *Predicted probability of csPCa as a function of PSAD across selected prostate volumes (left). PSAD threshold required to achieve predefined risk probabilities of 25 %, 40 % and 50 % as a function of prostate volume (right). Both panels derived from Model C (logistic regression with log-PSAD and B-spline volume terms including their non-linear interaction). The grey dotted line indicates the conventional PSAD threshold of 0.15 ng/ml/cm³.*

# S4 — Decision curve analysis

### Methods

Decision curve analysis (Vickers & Elkin 2006) was used to express diagnostic performance as net clinical benefit across a range of threshold probabilities (p_t = 0.05 to 0.50). Net benefit was computed as NB = (TP / N) − (FP / N) × (p_t / (1 − p_t)). Five strategies were compared: biopsy all, biopsy none, fixed PSAD ≥ 0.15 ng/ml/cm³, volume-stratified PSAD (0.25 / 0.15 / 0.12 ng/ml/cm³ for <30 / 30–50 / >50 cm³), and continuous prediction from Model C.

### Results

Strategy outcomes per volume stratum at the default cut-offs (Table S6):

| **Stratum** | **Strategy** | **Biopsies** | **csPCa detected** | **Missed** | **NNB** |
| --- | --- | --- | --- | --- | --- |
| Overall | Fixed 0.15 | 1,778 | 1,103 | 532 | 1.61 |
| Overall | Volume-stratified | 1,812 | 1,053 | 582 | 1.72 |
| <30 cm³ | Fixed 0.15 | 479 | 343 | 51 | 1.40 |
| <30 cm³ | Volume-stratified | 283 | 227 | 167 | 1.25 |
| 30–50 cm³ | Fixed 0.15 | 857 | 534 | 203 | 1.60 |
| 30–50 cm³ | Volume-stratified | 857 | 534 | 203 | 1.60 |
| >50 cm³ | Fixed 0.15 | 442 | 226 | 278 | 1.96 |
| >50 cm³ | Volume-stratified | 672 | 292 | 212 | 2.30 |

***Table S6.*** *Biopsy strategy outcomes per volume stratum. NNB = number needed to biopsy to detect one csPCa.*

Trade-offs between volume-stratified and fixed-0.15 strategy at p_t = 0.10 (Table S7):

| **Stratum** | **Δ biopsies** | **Δ csPCa detected** | **ΔNB at p_t = 0.10** |
| --- | --- | --- | --- |
| <30 cm³ | −196 (avoided) | −116 (missed) | −0.17 |
| 30–50 cm³ | 0 | 0 | ≈ 0 |
| >50 cm³ | +230 (additional) | +66 (additional) | +0.027 |
| >80 cm³ * | — | — | — |
| >100 cm³ * | — | — | — |
| >120 cm³ * | — | — | — |

***Table S7.*** *Trade-offs of the volume-stratified strategy relative to the fixed PSAD ≥ 0.15 cut-off. * Subgroup sizes preclude reliable net-benefit inference and are reported only for completeness.*

The volume-stratified strategy was inferior to the fixed cut-off in the <30 cm³ stratum at threshold probabilities ≤ 20 %, equivalent in the 30–50 cm³ stratum, and superior in the >50 cm³ stratum (ΔNB = +0.027 at p_t = 0.10). Continuous prediction from Model C dominated both threshold-based strategies across all examined threshold probabilities.

***Figure S3.*** *Decision curve analysis across the overall cohort. Left: net benefit of five biopsy strategies across threshold probabilities (p_t) from 0.05 to 0.50. Right: corresponding net biopsies avoided per 100 patients.*

***Figure S4.*** *Decision curve analysis stratified by prostate volume (<30, 30–50 and >50 cm³). Each panel shows the net benefit of biopsy-all (grey dashed), biopsy-none (black dotted), fixed PSAD ≥ 0.15 (red), and volume-stratified PSAD (green) across threshold probabilities from 0.05 to 0.50.*

# S5 — Influence of chronic prostatitis on PSAD performance

### Methods

Chronic prostatitis was defined as histologically confirmed significant chronic inflammation in benign prostate tissue. Three complementary analyses were performed: (1) prostatitis was added to the multivariable logistic regression (log-PSAD + volume splines + age + prostatitis) with cluster-robust standard errors; (2) a likelihood-ratio test compared models with and without prostatitis; (3) the apparent ROC analysis was repeated after excluding all prostatitis cases.

### Results

Prostatitis was present in 1,556 of 3,923 sessions ([recompute] %). The prevalence rose with increasing prostate volume.

Multivariable association of prostatitis with csPCa (Table S8):

| **Analysis** | **OR (95 % CI)** | **p-value** | **LRT χ² (df)** |
| --- | --- | --- | --- |
| Logistic regression (log-PSAD + volume splines + age + prostatitis) | 0.39 (0.32–0.48) | < 0.001 | — |
| LRT: adding prostatitis to model | — | < 0.001 | 140.66 (1) |

***Table S8.*** *Adjusted association of chronic prostatitis with clinically significant prostate cancer.*

Sensitivity analysis with prostatitis cases excluded (Table S9):

| **Stratum** | **n (no prostatitis)** | **AUC** | **Cut-off** | **Full-sample AUC** | **Δ AUC** |
| --- | --- | --- | --- | --- | --- |
| Overall | 2,402 | 0.766 | 0.157 | 0.758 | +0.008 |
| <30 cm³ | 482 | 0.705 | 0.183 | 0.717 | −0.012 |
| 30–50 cm³ | 1,028 | 0.737 | 0.125 | 0.734 | +0.002 |
| >50 cm³ | 892 | 0.753 | 0.142 | 0.702 | +0.051 |
| >80 cm³ | 301 | 0.743 | 0.131 | 0.658 | +0.078 |
| >100 cm³ | 141 | 0.702 | 0.124 | 0.627 | +0.060 |
| >120 cm³ | 82 | 0.671 | 0.263 | 0.609 | +0.043 |

***Table S9.*** *Diagnostic performance of PSAD after excluding all chronic prostatitis cases.*

Chronic prostatitis was strongly negatively associated with csPCa after adjustment for PSAD, volume, and age (OR 0.39, 95 % CI 0.32–0.48). Excluding prostatitis cases substantially improved PSAD discrimination in larger glands (AUC +0.051 in >50 cm³ and +0.078 in >80 cm³), indicating that chronic inflammation is a relevant confounder of PSAD interpretation, particularly when prostate volume is large.

# S6 — PSAD performance in EAU-defined large-volume subgroups

### Methods

To examine PSAD discrimination at volumes beyond the conventional clinical cut-off, three exploratory subgroups were defined according to the EAU guideline definition of large prostates: >80, >100 and >120 cm³. These analyses extend the primary stratification of <30, 30–50 and >50 cm³ presented in the main manuscript. Performance estimates are taken from the bootstrap analysis described in Section S1 (Table S1).

### Results

PSAD discrimination decreases monotonically with increasing prostate volume. Corrected AUC drops from 0.658 (>80 cm³) to 0.627 (>100 cm³) to 0.609 (>120 cm³). Cut-off confidence intervals widen substantially in the largest subgroups, reflecting both reduced sample size and reduced discrimination.

Sensitivity of the conventional fixed PSAD ≥ 0.15 cut-off was 33.1 % at >80 cm³, 28.8 % at >100 cm³, and 33.3 % at >120 cm³ (Table S2). Combined with the wide cut-off confidence intervals reported in Table S1, these results indicate that PSAD-based risk assessment becomes structurally unreliable in very large prostates and that fixed-threshold strategies are particularly poorly suited to this subgroup.
